# Reciprocal activation between M1 macrophages and trophoblasts through CXCL9/STAT1/ZEB1/CCL2 axis promotes recurrent spontaneous abortion

**DOI:** 10.3389/fimmu.2025.1629370

**Published:** 2025-11-07

**Authors:** Sisi Yan, Xiang Wang, Qiuji Wu, Jinli Ding, Hui Qiu

**Affiliations:** 1 Department of Radiation and Medical Oncology, Hubei Key Laboratory of Tumor Biological Behaviors, Hubei Cancer Clinical Study Center, Zhongnan Hospital of Wuhan University, Wuhan, China; 2 Reproductive Medical Center, Renmin Hospital of Wuhan University and Hubei Clinic Research center for Assisted Reproductive Technology and Embryonic Development, Wuhan, China

**Keywords:** recurrent spontaneous abortion, M1-Mφ, trophoblasts, CXCL9, migration and invasion, EMT

## Abstract

**Background:**

The crosstalk between macrophages and trophoblasts plays a crucial role in the development and progression of recurrent spontaneous abortion (RSA). Although M1 macrophages (M1-Mφ) are known to accumulate in RSA decidual tissues, their direct functional impact on trophoblasts remains poorly characterized.

**Methods:**

We established an M1-Mφ-trophoblast coculture system to investigate this interaction. CXCL9 expression was quantified in clinical samples and cell lines using qPCR, ELISA, and immunofluorescence. The migration and invasion capacities of trophoblasts were evaluated through wound healing and Transwell assays. A series of rescue experiments were conducted to uncover the underlying mechanism. Finally, an *in vivo* animal model was carried out to validate the corresponding functions of the CXCL9-related axis.

**Results:**

Our results revealed that M1-Mφ inhibited the migration and invasion of trophoblasts by releasing CXCL9. The expression of CXCL9 in decidual tissues was significantly increased in RSA samples compared to healthy controls. Mechanistically, CXCL9 activated the CXCR3-dependent JAK/STAT1 signaling pathway. Activated STAT1 induced transcriptional upregulation of ZEB1 via IRF1, which in turn promoted the release of CCL2 to enhance macrophage recruitment. *In vivo*, inhibition of CXCL9 reduced embryo resorption in LPS-induced abortion mice, attenuated macrophage infiltration, and restored trophoblast migration and invasion.

**Conclusion:**

Our work identifies a novel mechanism by which M1-Mφ regulate trophoblast migration and invasion through the CXCL9/STAT1/IRF1/ZEB1 axis, which in turn leads to the release of CCL2 that promotes macrophage infiltration in RSA, highlighting a new form of crosstalk between macrophages and trophoblasts.

## 1 Introduction

Recurrent spontaneous abortion (RSA) is clinically defined as the loss of two or more pregnancies before 20 weeks of gestation, affecting 2-5% of reproductive-aged women worldwide (1). The etiology of RSA is multifactorial and heterogeneous, encompassing chromosomal abnormalities, immune dysregulation, endocrine disorders, as well as environmental and lifestyle influences (2–4). Accumulating evidence also associates immune-mediated gynecological conditions such as endometriosis with an elevated risk of pregnancy loss, implying that shared inflammatory and immunologic mechanisms may underlie disrupted embryo implantation and placental development (5, 6). Nevertheless, the precise pathogenic mechanisms remain incompletely elucidated, warranting further investigation.

During early pregnancy, extravillous trophoblasts (EVTs) play pivotal roles at the maternal-fetal interface by invading the endometrial stroma and remodeling the spiral uterine arteries (7). Epithelial-mesenchymal transition (EMT), characterized by increased cell motility and invasive potential, plays a critical role in regulating trophoblast migration and invasion (8, 9). Simultaneously, the establishment of precise immune tolerance at the maternal-fetal interface is crucial for successful pregnancy maintenance (10). This delicate immunological balance is maintained through sophisticated cellular crosstalk among trophoblasts, immune cells, and decidual stromal cells. Disruptions in this equilibrium may lead to various pregnancy complications, including RSA and preeclampsia (11, 12).

Among the diverse immune cell populations in decidual tissue, macrophages are particularly noteworthy, accounting for 20-30% of first-trimester leukocytes and playing multifaceted roles in pregnancy maintenance (13). Notably, accumulating evidence from our group and others has demonstrated abnormal accumulation of pro-inflammatory M1 macrophages (M1-Mφ) in RSA decidua, implicating their pathological contribution to pregnancy loss (14–16). Macrophages are known to shape the local microenvironment through paracrine signaling, thereby influencing trophoblast behavior (17, 18). Our previous work has specifically shown that M1-Mφ-derived extracellular vesicles can impair trophoblast migration and invasion, potentially driving RSA pathogenesis (19). While extracellular vesicles represent one important communication mechanism, cytokines and chemokines serve as equally crucial mediators of intercellular crosstalk. However, the specific roles of these soluble factors in M1-Mφ-trophoblast communication during RSA remain poorly understood.

Our study reveals a novel regulatory axis wherein M1-Mφ-derived CXCL9 impairs trophoblast migration and invasion through activation of the JAK/STAT1 signaling pathway. Mechanistically, we demonstrate that ZEB1 mediates STAT1-dependent EMT modulation in trophoblasts by interacting with IRF1, which in turn promotes the generation of CCL2 to facilitate macrophage recruitment. This reciprocal crosstalk between trophoblasts and macrophages establishes a positive feedback loop that exacerbates the pathological microenvironment at the maternal-fetal interface, ultimately contributing to RSA progression.

## 2 Materials and methods

### 2.1 Patients and tissue samples

A brief summary of studied molecules is provided in **Supplementary Table S1**. Between September 2021 and February 2022, villous and decidual tissue samples of induced abortion (control group, n=10) and RSA (RSA group, n=10) were obtained from Renmin Hospital of Wuhan University. RSA was identified as the sequential loss of two or more pregnancies before 20 weeks of pregnancy. Exclusion criteria were as follows, (1) endocrine or metabolic diseases (such as thyroid dysfunction, PCOS, diabetes mellitus, or diabetes mellitus), (2) karyotype abnormalities, (3) uterine abnormalities, or (4) infections (e.g., HIV, TORCH, or syphilis). Baseline data of the included patients are listed in **Supplementary Table S2**. The study was approved by the Institutional Review and Ethics Boards of Renmin Hospital of Wuhan University (Ethical Approval Number: WDRY2021-K044).

All tissue collection procedures were performed in a sterile environment, then the tissues were placed in a pre-cooled container filled with sterile PBS containing 1% penicillin-streptomycin during transport. The freshly obtained placental tissues were rinsed three times with sterile PBS and divided for parallel processing: one portion was embedded for histological and immunofluorescence analyses, and the other portion was snap-frozen at −80°C for subsequent qPCR and Western blot assays.

### 2.2 Animals and experimental protocol

Eight‐week‐old female C57BL/6 and male Balb/c mice were maintained in a specific pathogen-free (SPF) environment under standard environmental conditions. After adaptive feeding, the female mice were mated with the male mice at a ratio of 2:1. The day when a vaginal plug became visible was defined as embryonic day 0.5 (E0.5). Lipopolysaccharide (LPS; 0.25 mg/kg, Sigma) was intraperitoneally injected on the afternoon of E7.5 to induce abortion, and the control group was injected with saline solution. In the anti-CXCL9 group, anti-Mouse CXCL9/MIG Antibody (1 mg/kg; MCE, Shanghai) were injected into female C57BL/6 mice by intravenously administered at 8:00 am on E7.5, E10.5 and E13.5. All female mice were euthanized by isoflurane anesthesia on E13.5. The embryo resorption rate of each mouse was calculated to assess pregnancy outcome and was defined as the number of resorbed embryos/(number of resorbed embryos + number of fetuses surviving) × 100%. All protocols and experiments were approved by the Animal Care and Use Committee of the Wuhan University (Ethical Approval Number: 20190710).

### 2.3 Cell culture and treatments

The trophoblast cell line HTR-8/SVneo (HTR-8) and the human monocyte cell line THP-1 were grown in RPMI-1640 medium (Gibco) supplemented with 10% fetal bovine serum (FBS) (Gibco) at 37°C in 5% CO_2_. THP-1 cells were differentiated into M0 macrophages by treatment with 100 ng/mL phorbol 12-myristate 13-acetate (PMA; Sigma-Aldrich, USA) for 24 h. To induce M1-Mφ, M0 macrophages were stimulated with 100 ng/mL lipopolysaccharide (LPS; Sigma) plus 20 ng/mL IFN-γ (PeproTech, USA) (20). In the co-culture model, macrophages were incubated in the upper chambers, and HTR-8 was placed into the lower chamber of each insert, then analyzed after 48 h of co-culture.

The siRNA sequences targeting STAT1, IRF1, and ZEB1 are listed in Additional file 1: **Supplementary Table S3** and synthesized by Vigene (Shandong, China). HTR-8 cells were transfected with indicated siRNA or control using Lipofectamine 2000 reagent (Invitrogen, CA, USA) according to the manufacturer’s instructions.

For the treatment of CXCL9, 50 ng/ml CXCL9 (PeproTech) or 1.0 μg/ml anti-human CXCL9 neutralizing antibody (anti-CXCL9, PeproTech) was added to HTR-8 cells. For CCL2 treatment, M0-Mφ were treated with 50 ng/mL CCL2 (PeproTech).

### 2.4 Enzyme-linked immunosorbent assay

The culture supernatants were collected, and the concentrations of CXCL9 and CCL2 in the supernatants were quantified using commercially available sandwich ELISA kits (R&D Systems; USA) according to the manufacturer’s protocols. All samples were assayed in triplicate, and the absorbance was measured at 450 nm with wavelength correction at 540 nm using a microplate reader (BioTek Instruments, Winooski, VT, USA). Standard curves were generated for each assay using recombinant human cytokines provided with the kits.

### 2.5 Quantitative polymerase chain reaction

Total RNA was isolated from cells using TRIzol reagent (Accurate Biology, China) according to the manufacturer’s instructions. cDNA was synthesized using the PrimeScript RT reagent kit (Accurate). PCR was performed with 7500 Real-Time PCR system (Applied Biosystems, Foster City, CA, USA). The 2^−ΔΔ^ Ct method was used to relatively quantify the levels of gene expression. Primers used to measure mRNA expression levels are shown in **Supplementary Table S4**. Each sample was analyzed in triplicate.

### 2.6 Western blot analysis

The RIPA lysis buffer (Beyotime, China) was used to extract proteins from cells and tissues. Protein samples were separated by 10% SDS-PAGE gel and transferred onto a PVDF membrane. The membranes were incubated overnight at 4°C with the primary antibodies against Actin (Proteintech, Cat# 20536-1-AP, 1:5000), CXCL9 (Cat# 22355-1-AP, 1:1000), CXCR3 (Cat# 26756-1-AP, 1:1000), E-cadherin (Cat# 20874-1-AP, 1:5000), N-cadherin (Cat# 22018-1-AP, 1:3000), Vimentin (Cat# 10366-1-AP, 1:5000), STAT1 (Cat# 10144-2-AP, 1:3000), p-STAT1 (Cat# 28977-1-AP, 1:1000), IRF1 (Cat# 11335-1-AP, 1:500), ZEB1 (Cat# 21544-1-AP, 1:1000), JAK1 (Abmart, Cat# TA5012, 1:1000), p-JAK1 (Cat# TP56310, 1:1000), JAK2 (Cat# T55287, 1:1000), p-JAK2 (Cat# T56570, 1:1000. After washing, membranes were incubated with the secondary antibody (Proteintech) for 1 h at room temperature. Protein bands were visualized using an ECL system (Bio-Rad, Hercules, CA, USA).

### 2.7 Immunohistochemistry

IHC was performed on paraffin-embedded tissue sections according to established protocols (21). Briefly, sections were incubated with primary antibodies against CXCL9, E-cadherin, Vimentin, CD86, p-STAT1, IRF1 and ZEB1 overnight at 4°C, followed by appropriate secondary antibodies. After DAB development and hematoxylin counterstaining, images were captured using a light microscope (Olympus, Japan), and immunohistochemistry was scored based on the intensity of staining and the proportion of positive cells.

### 2.8 Immunofluorescence

After permeabilization, tissue sections and cultured cells were incubated overnight at 4°C with the following primary antibodies rabbit anti-CXCL9, anti-CXCR3, anti-CD68, anti-CD86, anti-E-cadherin anti-N-cadherin and anti-CCL2 (Proteintech). After PBS washes, samples were counterstained with 4′,6-diamidino-2-phenylindole (DAPI) nucleic acid stain (Invitrogen) for 5 min at room temperature and mounted with antifade medium. Fluorescent images were captured using fluorescence microscopy (Olympus, Japan).

### 2.9 Wound healing assay

For wound healing experiments, HTR-8 cells (5×10^5^) were seeded in 6-well plates and incubated overnight to 80-90% confluence. The cells were scratched with a 1 mL pipette tip and then washed with PBS. The wound area was determined using an inverted microscope at 0 h and 24 h post-scratching to evaluate cell migration.

### 2.10 Transwell invasion assay

Trophoblast cell invasion was assessed using Matrigel-coated Transwell chambers (8 μm pore size; Corning) in 24-well plates. Briefly, 5×10^4^ HTR-8 cells were seeded on the upper chamber coated with Matrigel (1:8 dilution; Sigma, St Louis, MO), and polarized M1-Mφ were placed in the lower chamber. After 24 h of incubation, cells that penetrated the membrane were fixed with formaldehyde and quantified by crystal violet staining.

### 2.11 Luciferase reporter assay

The treated HTR-8 cells were co-transfected with ZEB1 luciferase reporter and Renilla luciferase plasmid using Lipofectamine 2000 transfection reagent (Invitrogen, USA). After 48 h of the transfection, the luciferase activity was measured using the Dual Luciferase Reporter Gene Assay Kit (Yeasen, China) according to the manufacturer’s protocols. Luciferase activity was normalized to the corresponding Renilla luciferase activity for each sample to account for transfection efficiency.

### 2.12 Chromatin immunoprecipitation assay

Four pairs of primers targeting the ZEB1 promoter were synthesized by Sangon Biotech (Shanghai, China), and the sequences of the primers were applied in **Supplementary Table S5**. ChIP assay was conducted with the ChIP Kit (ABclonal Technology, China) according to the manufacture’s instruction. In brief, cells (1×10^7^) were fixed with 1% formaldehyde to crosslink for 10 min at room temperature, and then treated with 125 mM glycine to quench the crosslinking. Chromatin extracts containing DNA fragments were immunoprecipitated using IRF1 antibody or normal rabbit IgG overnight. After washing and reverse crosslinking, the DNA fragments were purified and amplified by qPCR. Data were normalized to input DNA and presented as fold enrichment relative to IgG control.

### 2.13 Statistical analysis

Statistical analyses were performed using SPSS 22.0 software (IBM SPSS, Chicago, USA). Quantitative data are presented as mean ± SD from three independent experiments. Differences between groups were assessed using Student’s t test (for two groups) or one-way ANOVA with Tukey’s multiple comparison test (for multiple groups). Correlation analysis was performed by Spearman’s correlation coefficient. *P* < 0.05 was considered statistically significant.

## 3 Results

### 3.1 M1-Mφ-derived CXCL9 impairs trophoblasts invasion and migration

**Figure 1:**
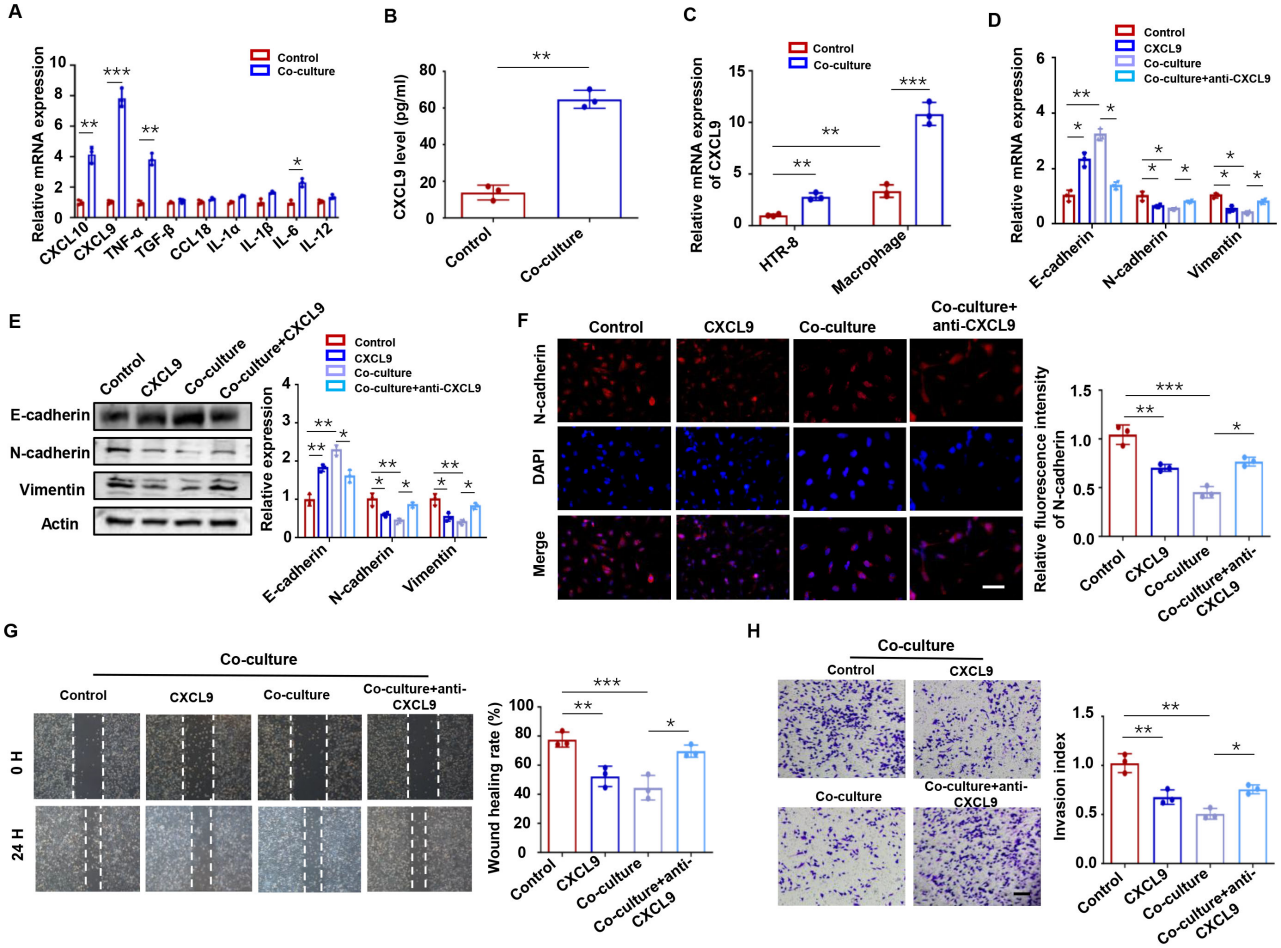
M1-Mφ-derived CXCL9 impairs trophoblasts invasion and migration. **(A)** mRNA expression levels in HTR-8 cells cultured alone or co-cultured with M1-Mφ. **(B)** ELISA assays of CXCL9 in the supernatant of M1-Mφ alone or co-cultured with HTR-8 cells. **(C)** CXCL9 mRNA expression in HTR-8 and M1-Mφ with or without 48 h of co-culture. **(D-F)** The expression of EMT markers in HTR-8 cells alone, CXCL9-supplemented HTR-8 cells, M1-Mφ-co-cultured HTR-8 cells, and anti-CXCL9 M1-Mφ-co-cultured HTR-8 cells were analyzed by qPCR, western blot and immunofluorescence. Scale bar: 20 μm. **(G, H)** Migration and invasion of anti-CXCL9 M1-Mφ-co-cultured HTR-8 cells and its control were measured by wound-healing assay and transwell assays, respectively. n = 3, Scale bar: 50 μm; ^*^P < 0.05, ^**^P < 0.01, ^***^P < 0.001.

To investigate the effects of M1-Mφ on trophoblast function, we established a co-culture system of M1-Mφ with HTR-8 trophoblast cells (**Supplementary Figure S1A**). After 48 h of co-culture, M1-Mφ induced EMT marker changes in trophoblasts, characterized by upregulation of E-cadherin and downregulation of N-cadherin (**Supplementary Figures S2A, B**). These alterations correlated with significantly impaired migratory and invasive capacities compared to controls (**Supplementary Figures S2C, D**). Given the established role of macrophage-derived soluble factors in modulating trophoblast function (22), we screened EMT-related cytokines/chemokines and identified CXCL9 as the most significantly upregulated factor in co-cultured supernatants versus control groups (**Figure 1A**). ELISA analysis confirmed increased CXCL9 secretion in co-culture supernatants (**Figure 1B**). The basal level of CXCL9 was much higher in M1-Mφ than in HTR-8 cells, and co-culture selectively promoted CXCL9 expression in M1-Mφ but not trophoblasts (**Figure 1C**). Based on these findings, we hypothesized that M1-Mφ might affect trophoblast function via CXCL9.

To validate the functional role of CXCL9 in trophoblasts, we treated HTR-8 cells with recombinant CXCL9. Our results showed that CXCL9 treatment upregulated E-cadherin while downregulating N-cadherin and Vimentin expression in HTR-8 cells compared to untreated controls. Conversely, the administration of a CXCL9 neutralizing antibody in the co-culture system reversed these effects. (**Figures 1D, E**). These findings were further corroborated by immunofluorescence staining of N-cadherin (**Figure 1F**). Functional assays demonstrated that CXCL9 significantly inhibited trophoblast migration and invasion relative to control conditions, whereas CXCL9 neutralization restored these capacities (**Figures 1G, H**). These data indicate that M1-Mφ regulate trophoblast abilities through CXCL9-mediated EMT modulation.

### 3.2 Aberrant expression of CXCL9 in RSA placental tissues

**Figure 2:**
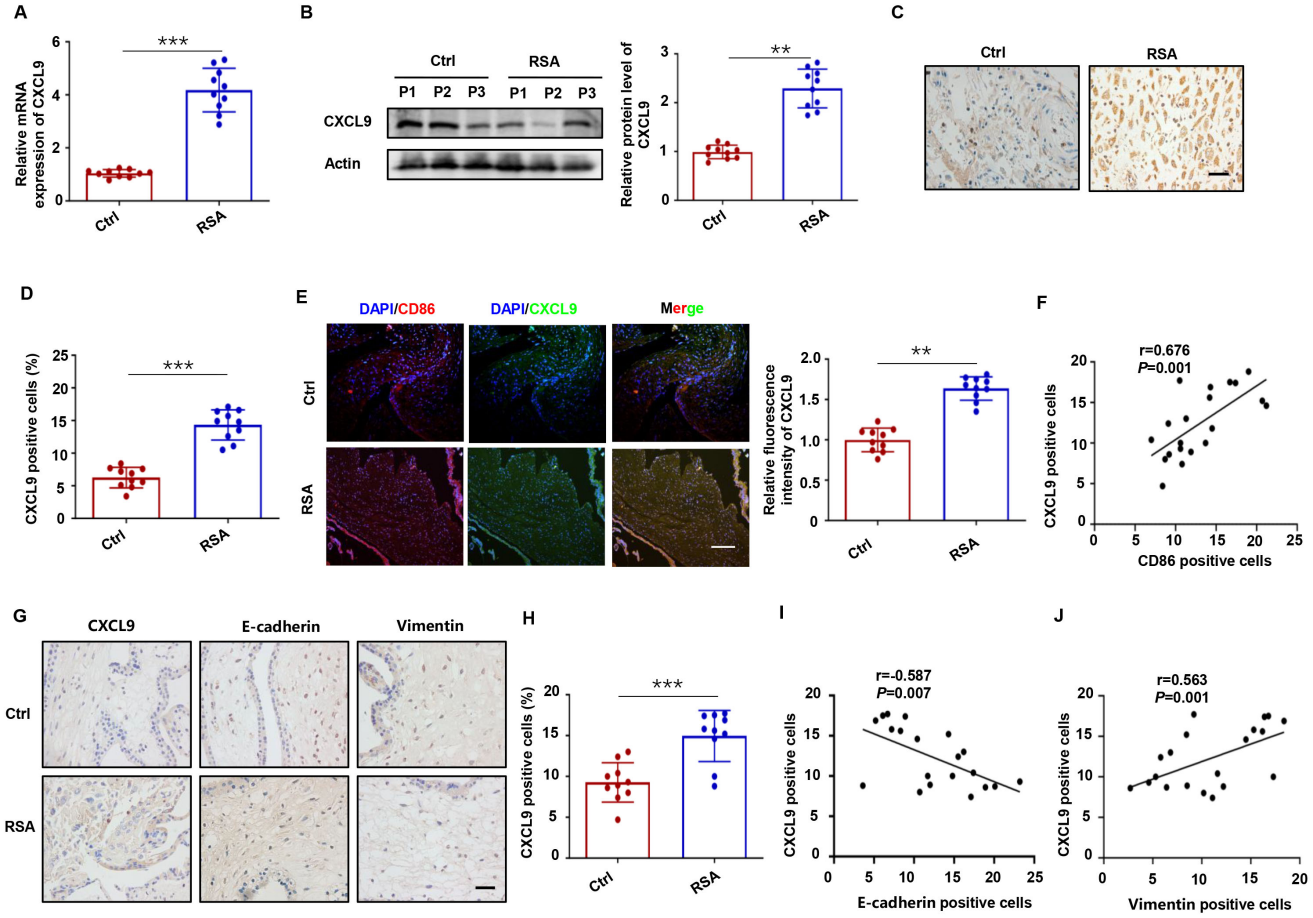
Aberrant expression of CXCL9 in RSA placental tissues. **(A, B)** The mRNA and protein levels of CXCL9 in decidual tissues of normal and RSA patients. **(C, D)** The distribution of CXCL9 in decidual tissues of normal and RSA patients was detected by IHC. Scale bars: 20 μm. **(E)** The distribution of CXCL9 in CD86^+^ macrophages were analyzed by immunofluorescence. Scale bar: 100 μm. **(F)** The correlation between the proportion of CD86^+^ and CXCL9^+^ cells in decidual tissues of normal and RSA patients. **(G)** IHC analysis of the CXCL9, E-cadherin and Vimentin expression in the placental villous tissues of normal and RSA patients. **(H)** IHC analysis of CXCL9 positive cells in villous tissues of normal and RSA patients. **(I)** Correlation analysis between levels of CXCL9 and E-cadherin in the placental villous tissues of normal and RSA patients. **(J)** Correlation analysis between levels of CXCL9 and Vimentin in the placental villous tissues of normal and RSA patients. ^**^P < 0.01, ^***^P < 0.001.

We next examined CXCL9 expression patterns in placental villous and decidual tissues from normal pregnancies and RSA patients, and the results revealed significantly elevated CXCL9 mRNA and protein levels in RSA decidual tissues compared to controls (**Figures 2A-D**). In addition, CXCL9 protein expression correlated with miscarriage history (**Supplementary Figure S3**). Double immunofluorescence analysis demonstrated co-localization of CD86 (an M1 macrophage marker) and CXCL9 in decidual tissues, with significantly intensified CXCL9 expression in RSA samples (**Figure 2E**). A strong positive correlation between CD86^+^ and CXCL9^+^ cells was observed (r = 0.676, P = 0.001; **Figure 2F**), indicating that the aberrant expression of CXCL9 in M1-Mφ might contribute to RSA pathogenesis. IHC analysis of villous tissues demonstrated increased CXCL9 positive cells infiltration in RSA patients, accompanied by elevated E-cadherin and decreased Vimentin expression (**Figures 2G, H**). To further explore the relationship between CXCL9 and the EMT process, correlation analysis revealed a statistically significant negative correlation between CXCL9 and E-cadherin expression (r = −0.587, P = 0.007; **Figure 2I**), while a significant positive correlation between the expression levels of CXCL9 and Vimentin (r = 0.563, P = 0.001; **Figure 2J**). These findings indicate that dysregulated CXCL9 expression in placental villous and decidual tissues may be associated with the pathogenesis of RSA.

### 3.3 CXCL9-CXCR3 axis mediates trophoblast invasion and migration

**Figure 3:**
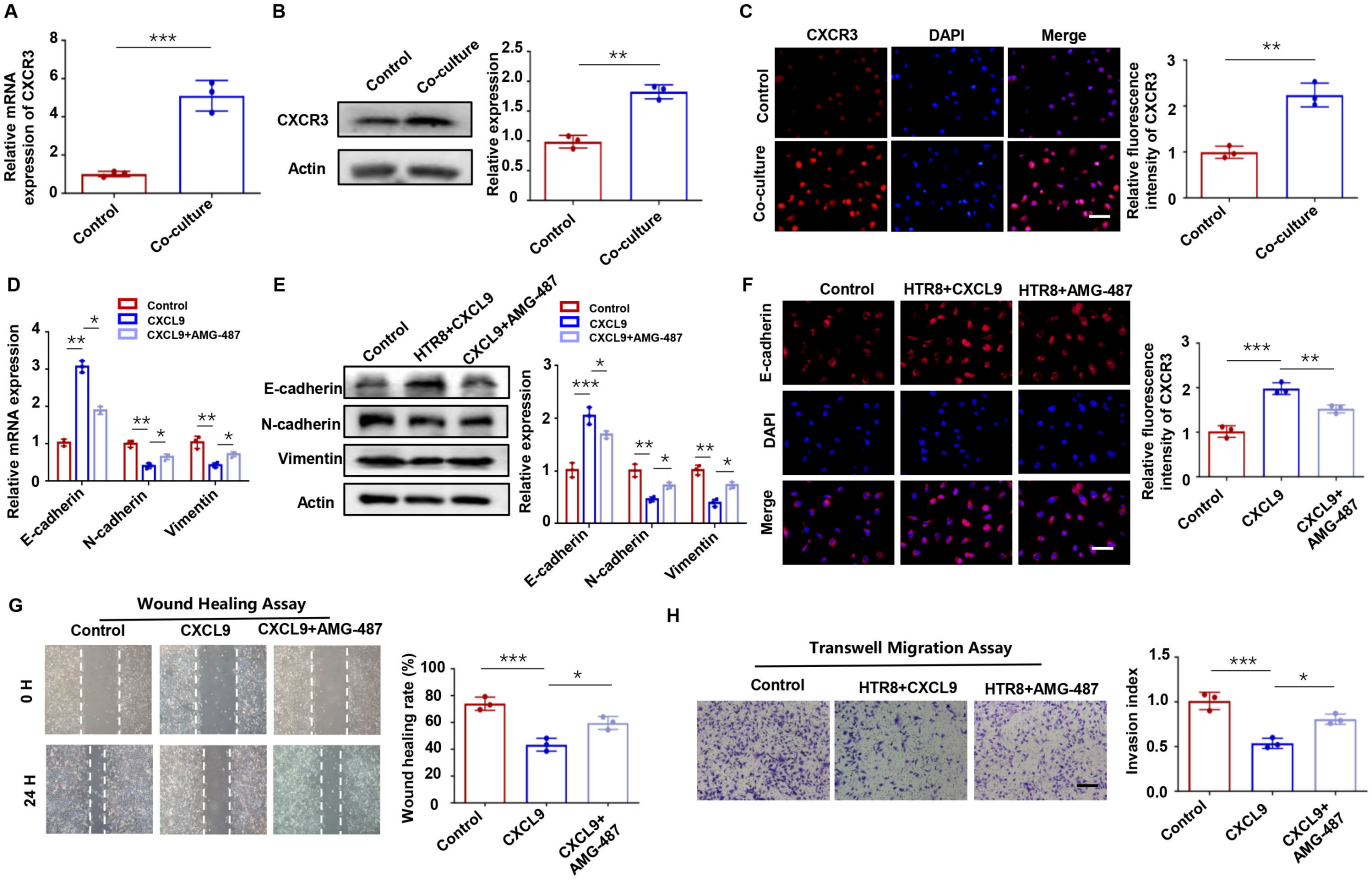
CXCL9-CXCR3 axis mediates trophoblast invasion and migration. **(A-C)** CXCR3 expression in HTR-8 cells with or without M1-Mφ coculture, as assessed by qPCR, Western blot, and immunofluorescence. **(D-F)** Expression of EMT markers in HTR-8 cells alone, CXCL9-treated HTR-8 cells and HTR-8 cells co-treated with CXCL9 and AMG-487 were analyzed by qPCR, western blot and immunofluorescence analysis. **(G, H)** Migration and invasion of abovementioned groups were measured by wound-healing assay and transwell assays, respectively. n = 3, Scale bar: 20 μm; ^*^P < 0.05, ^**^P < 0.01, ^***^P < 0.001.

Chemokine receptor CXCR3, the cognate receptor for CXCL9, is a well-characterized mediator of immune and inflammatory responses (23, 24). Our results showed CXCR3 activation in HTR-8 cells after M1-Mφ co-culture (**Figure 3A**), as demonstrated by western blot and immunofluorescence staining (**Figures 3B, C**). To elucidate the underlying mechanism, we employed the specific CXCR3 antagonist AMG 487. The results indicated that AMG 487 pretreatment reversed CXCL9-induced upregulation of E-cadherin and downregulation of N-cadherin and Vimentin (**Figures 3D-F**). Functional assays provided compelling evidence that CXCL9’s inhibitory effects on trophoblast migration and invasion were primarily dependent on CXCR3 signaling, as AMG 487 pretreatment reversed these phenotypic changes (**Figures 3G, H**). Therefore, M1-Mφ-derived CXCL9 regulates trophoblast motility primarily through specific activation of the CXCR3 receptor.

### 3.4 JAK/STAT1 pathway is downstream of CXCL9

**Figure 4:**
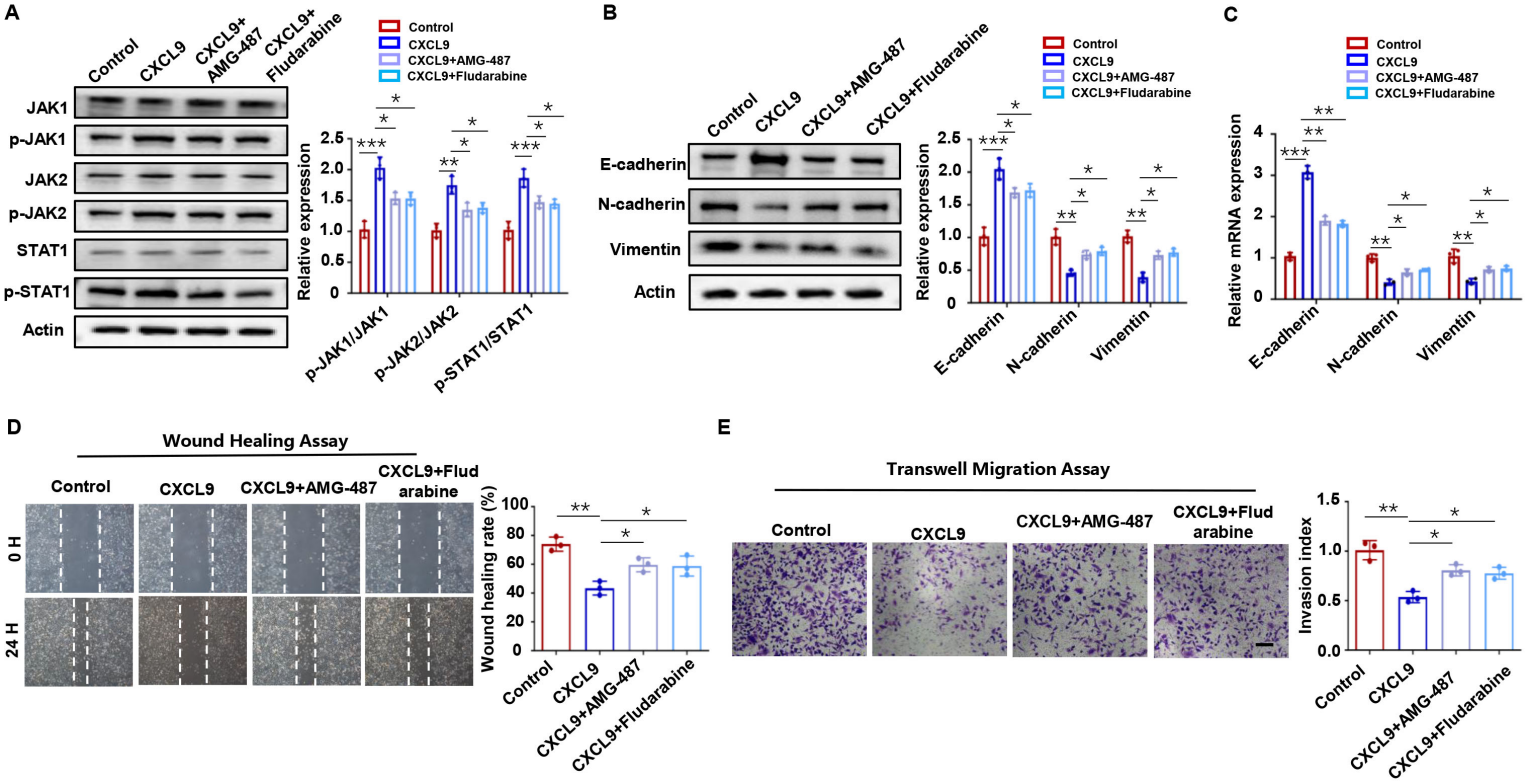
JAK/STAT1 pathway is downstream of CXCL9. **(A)** Western blot analysis of p-JAK1/JAK1, p-JAK2/JAK2 and p-STAT1/STAT1 in HTR-8 cells alone, CXCL9 treated HTR-8 cells, CXCL9+AMG-487 HTR-8 cells, and CXCL9+Fludarabine HTR-8 cells. **(B, C)** qPCR and Western blot analysis of E-cadherin, N-cadherin and Vimentin expression levels in the indicated groups. **(D, E)** Migration and invasion of indicated groups were detected by wound-healing assay and transwell assays, respectively. n = 3, Scale bar: 50 μm; ^*^P < 0.05, ^**^P < 0.01, ^***^P < 0.001.

The JAK/STAT pathway is a well-established downstream effector of chemokine signaling (25, 26). To determine whether CXCL9-CXCR3 axis activation triggers this pathway in trophoblasts, we performed systematic experiments. Western blot analysis revealed significant phosphorylation of JAK1/2 and STAT1 (p-JAK1/2, p-STAT1) upon CXCL9 stimulation (**Figure 4A**). In contrast, STAT3 phosphorylation remained unaffected (**Supplementary Figure S4**). Importantly, CXCR3 inhibition with AMG 487 completely abrogated CXCL9-induced JAK/STAT1 activation (**Figure 4A**). These results suggest that the JAK/STAT1 pathway may act as a downstream effector of the CXCL9-CXCR3 axis. To further validate this mechanism, we employed the STAT1-specific inhibitor fludarabine. We found that Fludarabine effectively blocked CXCL9-mediated JAK/STAT1 activation (**Figure 4A**), confirming STAT1 as a key downstream effector. Functional assays demonstrated that JAK/STAT1 pathway inhibition reversed CXCL9’s suppressive effects on trophoblast migration and invasion (**Figures 4B-E**). Hence, a preliminary conclusion would be that CXCL9 activates the CXCR3-JAK/STAT1 pathway to suppresse trophoblast invasion and migration.

### 3.5 CXCL9 activates JAK/STAT1 to regulate ZEB1

**Figure 5:**
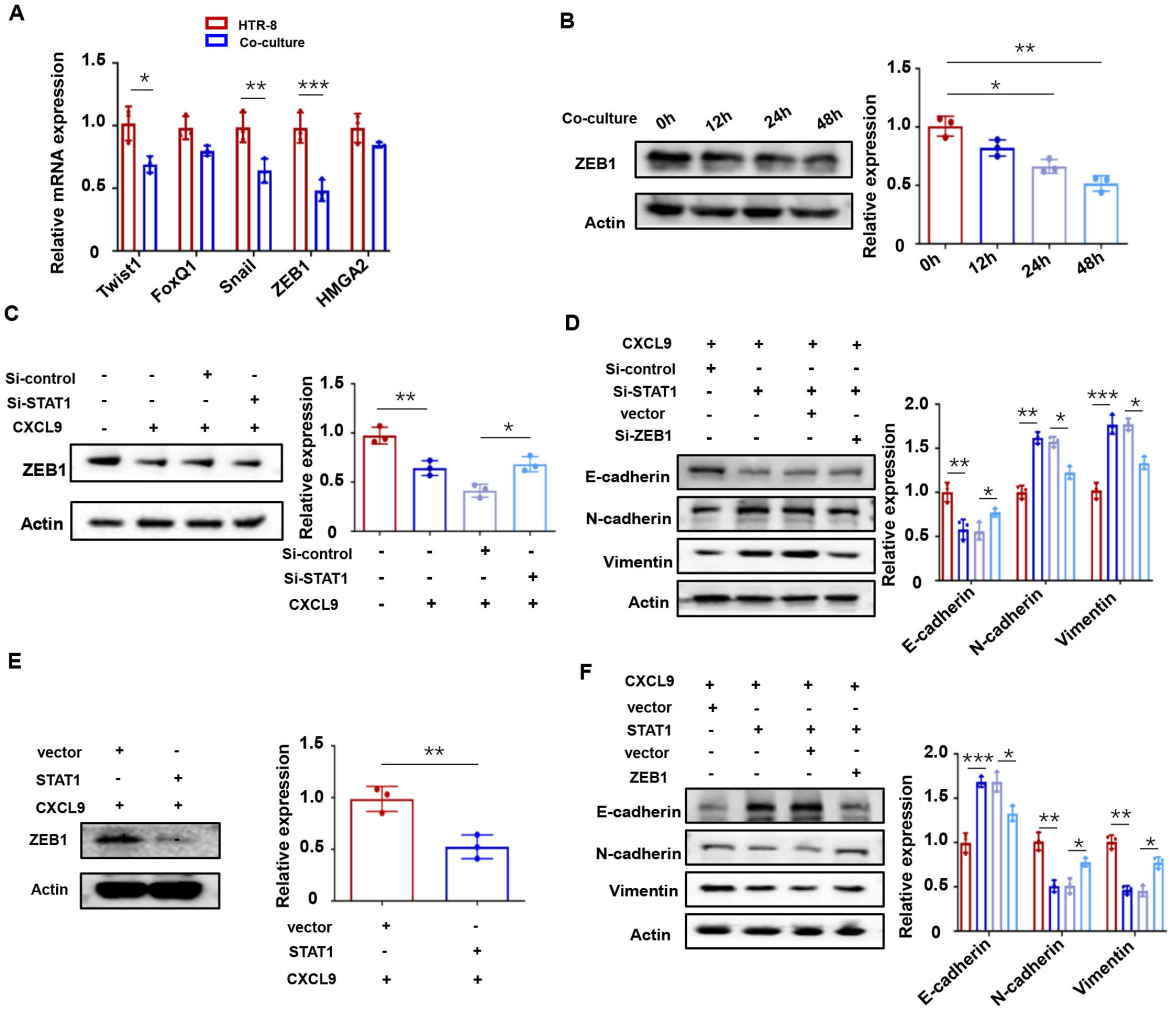
CXCL9 activates JAK/STAT1 to regulate ZEB1. **(A)** Relative expression levels of representative EMT related genes were measured in HTR-8 with or without 48 h of M1-Mφ co-culture as determined by qPCR. **(B)** Western blot analysis of ZEB1 in HTR-8 cells co-cultured with M1-Mφ for the indicated times. **(C)** Western blot analysis of ZEB1 in HTR-8 cells transfected with Si-STAT1 and incubated with CXCL9 for 48 h afterwards. **(D)** Western blot analysis of E-cadherin, N-cadherin and Vimentin expression in HTR-8 cells transfected with Si-STAT1 or Si-ZEB1. **(E)** Western blot analysis of ZEB1 in HTR-8 cells transfected with STAT1 overexpression vectors plus CXCL9 treatment. **(F)** Western blot analysis of E-cadherin, N-cadherin and Vimentin expression in HTR-8 cells transfected with STAT1 or ZEB1. n = 3, ^*^P < 0.05, ^**^P < 0.01, ^***^P < 0.001.

Epithelial-mesenchymal transition (EMT), a process regulated by specific transcription factors, plays a pivotal role in suppressing epithelial marker proteins (27). Given our observation that M1-Mφ influences trophoblast EMT, we examined the expression of key EMT-related transcription factors (Twist1, FoxQ1, ZEB1, Snail, and HMGA2) in HTR-8 cells co-cultured with M1-Mφ using qPCR. Among these factors, ZEB1 showed the most significant downregulation (**Figure 5A**), with time-dependent protein reduction confirmed by western blot (**Figure 5B**). Considering the concurrent activation of STAT1 and downregulation of ZEB1 in M1-Mφ-induced EMT, we hypothesized a potential link between STAT1 activation and ZEB1 suppression. Indeed, STAT1 knockdown effectively reversed the CXCL9-mediated reduction in ZEB1 expression (**Figure 5C**). Furthermore, STAT1 silencing restored the EMT process in trophoblasts (decreased E-cadherin and increased N-cadherin and vimentin expression), while ZEB1 knockdown produced opposite effects (**Figure 5D**). Conversely, STAT1 overexpression significantly reduced ZEB1 levels compared to controls (**Figure 5E**). At the same time, STAT1 overexpression enhanced E-cadherin and reduced N-cadherin and Vimentin expressions, while STAT1-influenced EMT changes were rescued by ZEB1 co-overexpression (**Figure 5F**). These results indicate that CXCL9 activates the JAK/STAT1 pathway to suppress ZEB1, thereby impairing EMT in trophoblasts.

### 3.6 IRF1 mediates CXCL9/STAT1-dependent ZEB1 suppression

**Figure 6:**
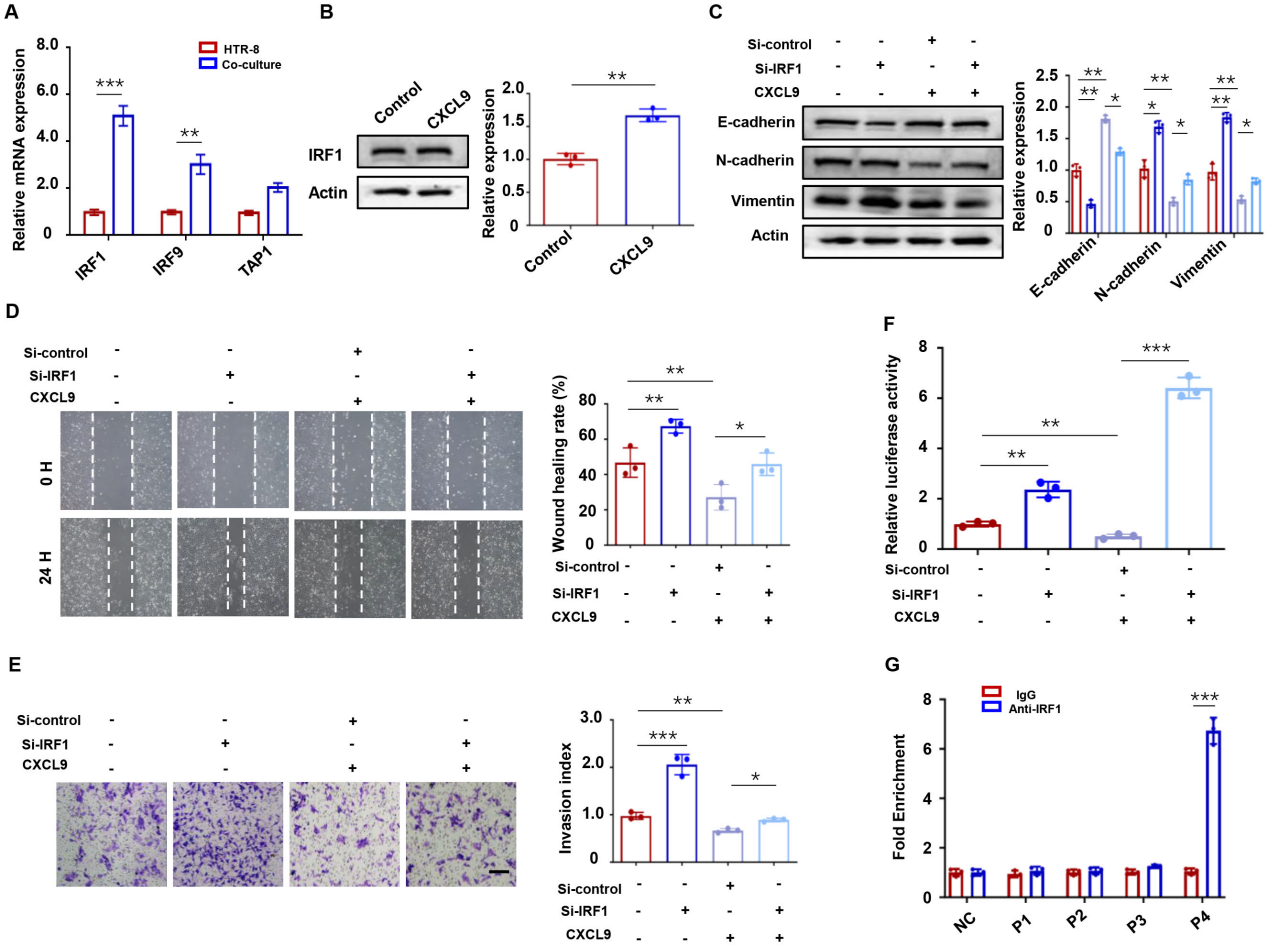
IRF1 mediates CXCL9/STAT1-dependent ZEB1 suppression. **(A)** qPCR analysis of STAT1 downstream target genes in HTR-8 cells co-cultured with M1-Mφ and HTR-8 cells alone. **(B)** IRF1 protein levels in HTR-8 cells co-cultured with M1-Mφ. **(C)** Western blot analysis of E-cadherin, N-cadherin and Vimentin expression in HTR-8 cells transfected with Si-IRF1 and incubated with CXCL9. **(D, E)** Migration and invasion of indicated groups were detected by wound-healing assay and transwell assays, respectively. **(F)** Luciferase reporter activity of the ZEB1 promoter in HTR-8 cells transfected with Si-control or Si-IRF1, followed by treatment with or without CXCL9. **(G)** ChIP assays showing the direct binding of IRF1 to the P4 region of the ZEB1 promoter. n = 3, Scale bar: 50 μm; ^*^P < 0.05, ^**^P < 0.01, ^***^P < 0.001.

To elucidate the mechanism of STAT1-mediated ZEB1 regulation in trophoblasts, we investigated potential downstream mediators of STAT1. Previous studies have reported that STAT1 transcriptionally regulates multiple targets, including IRF1, IRF9, and TAP1 (28, 29). CXCL9 treatment upregulated IRF1 at both the mRNA (**Figure 6A**) and protein levels (**Figure 6B**), implicating its role in ZEB1 suppression. To evaluate the functional relevance of IRF1 in trophoblast migration and invasion, we found that depletion of IRF1 resulted in a dramatic downregulation of E-cadherin and upregulation of N-cadherin and Vimentin (**Figure 6C**). Moreover, IRF1 depletion reversed the inhibitory effects of CXCL9 on HTR-8 cell migration and invasion (**Figures 6D, E**). To further dissect the mechanism by which IRF1 regulates ZEB1, we performed a luciferase reporter assay and observed that IRF1 knockdown increased ZEB1 promoter activity and rescued its suppression by CXCL9 (**Figure 6F**), suggesting that IRF1 directly represses ZEB1 transcription. Additionally, chromatin immunoprecipitation (ChIP) assays identified the P4 region of the ZEB1 promoter as a binding site for IRF1 (**Figure 6G**). These data collectively suggest that STAT1 regulates ZEB1 predominantly through IRF1-mediated transcriptional repression.

### 3.7 ZEB1-dependent CCL2 regulation promotes macrophage recruitment

**Figure 7:**
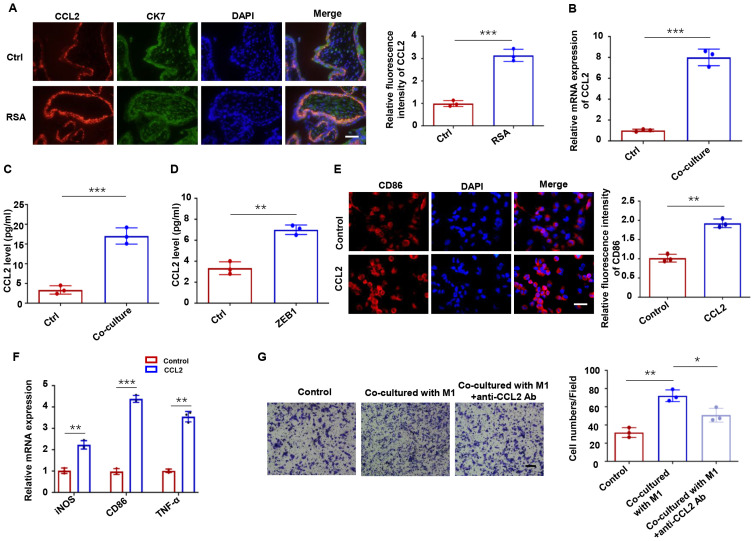
ZEB1-dependent CCL2 regulation promotes macrophage recruitment. **(A)** CCL2 expression in human placental villous tissues from RSA and healthy controls. **(B)** CCL2 mRNA and protein levels in HTR-8 cells co-cultured with M1-Mφ and HTR-8 cells alone. **(C)** ELISA assays of CCL2 protein secretion in HTR-8 cells co-cultured with M1-Mφ and HTR-8 cells alone. **(D)** ELISA assays of CCL2 in HTR-8 cells transfected with a ZEB1 overexpression plasmid or an empty vector control. **(E)** Immunofluorescence analysis of CD86 in macrophages with or without CCL2 treatment. **(F)** qRT-PCR of iNOS, CD86 and TNF-α in macrophages with or without CCL2 treatment. **(G)** Macrophage migration toward conditioned media with/without CCL2 neutralization. n = 3, Scale bar: 50 μm; ^*^P < 0.05, ^**^P < 0.01, ^***^P < 0.001.

The reciprocal regulation between trophoblasts and macrophages at the maternal-fetal interface is well documented, with trophoblast-derived cytokines playing pivotal roles in macrophage recruitment and polarization (30, 31). CCL2, a critical chemokine for macrophage recruitment at the maternal-fetal interface, has been implicated in this process (32). To investigate the role of CCL2 in RSA, we first examined its expression pattern in clinical specimens. Immunofluorescence analysis demonstrated significantly elevated CCL2 levels in CK7^+^ trophoblasts from RSA patients compared to healthy controls (**Figure 7A**). Consistent with these clinical findings, *in vitro* experiments showed that HTR-8 cells co-cultured with M1-Mφ exhibited increased CCL2 expression at both the mRNA (**Figure 7B**) and protein (**Figure 7C**) levels. We next explored whether ZEB1 modulates CCL2 production. ELISA assays revealed that ZEB1 overexpression upregulated CCL2 protein expression (**Figure 7D**). Additionally, immunofluorescence and qPCR analyses showed that CCL2 treatment promoted macrophage polarization toward the M1 phenotype (**Figures 7E, F**). Functional transwell assays demonstrated that HTR-8 cells educated by M1-Mφ enhanced THP-1 macrophage migration, an effect that was significantly attenuated by CCL2-neutralizing antibody treatment (**Figure 7G**). Taken together, our data reveal a novel positive feedback loop in which CXCL9 from M1 macrophages promotes trophoblast EMT via the STAT1/ZEB1 axis, while ZEB1-upregulated CCL2 from trophoblasts not only recruits macrophages but also enhances their M1 polarization.

### 3.8 Anti-CXCL9 treatment alleviates embryo resorption in mice

**Figure 8:**
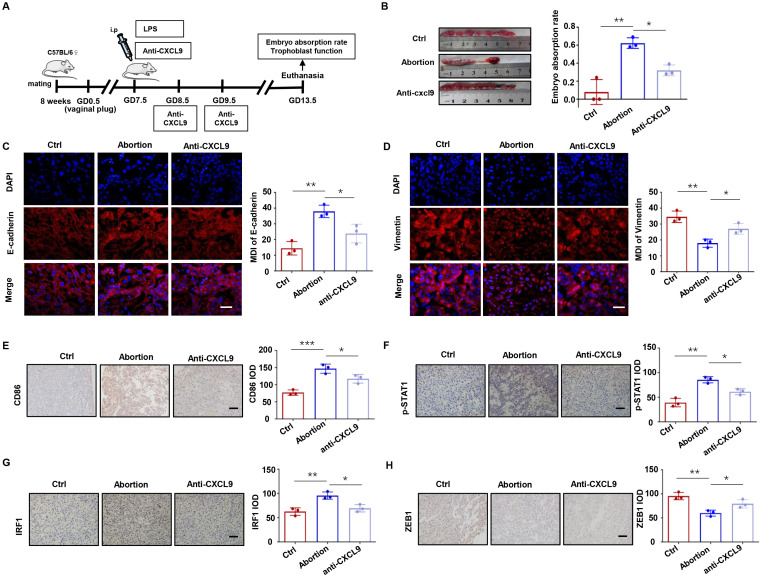
Anti-CXCL9 treatment alleviates embryo resorption rate in mice. **(A)** Experimental protocol for LPS-induced abortion model with anti-CXCL9 treatment. **(B)** The embryo resorption rates in the control, LPS-induced abortion and anti-CXCL9 groups. **(C, D)** Placental interface expression of E-cadherin and Vimentin. **(E)** IHC analysis of CD86 at the placental interface of mice. **(F-H)** IHC of p-STAT1, IRF1 and ZEB1 at the placental interface of mice. Scale bar, 50 µm. ^*^P < 0.05, ^**^P < 0.01, ^***^P < 0.001.

To further confirm the role of CXCL9 *in vivo*, we performed animal experiments using normal pregnant mice and LPS-induced abortion models with or without anti-CXCL9 neutralizing antibody treatment (**Figure 8A**). As shown in **Figure 8B**, anti-CXCL9 treatment significantly attenuated the embryo resorption rate in LPS-induced abortion models, suggesting its therapeutic potential. Immunofluorescence analysis revealed that the LPS-induced abortion group exhibited abnormally high E-cadherin and low Vimentin expression in placental tissues compared to normal pregnancy. Importantly, anti-CXCL9 treatment effectively reversed the expression of E-cadherin and Vimentin in the placentas of LPS-induced abortion mice (**Figures 8C, D**). We further investigated the immune microenvironment of the placenta. As indicated in **Figure 8E**, CD86^+^ cells in the placentas of the abortion group were significantly more abundant than in the normal group, whereas anti-CXCL9 treatment notably reversed this effect. Consistent with our *in vitro* findings, immunohistochemical staining confirmed that anti-CXCL9 treatment abolished STAT1 phosphorylation, downregulated IRF1 expression, and restored ZEB1 levels (**Figures 8F-H**). These data demonstrate that CXCL9 blockade could ameliorate embryonic resorption, restore trophoblast function, and attenuate macrophage recruitment in the LPS-induced abortion models.

## 4 Discussion

**Figure 9:**
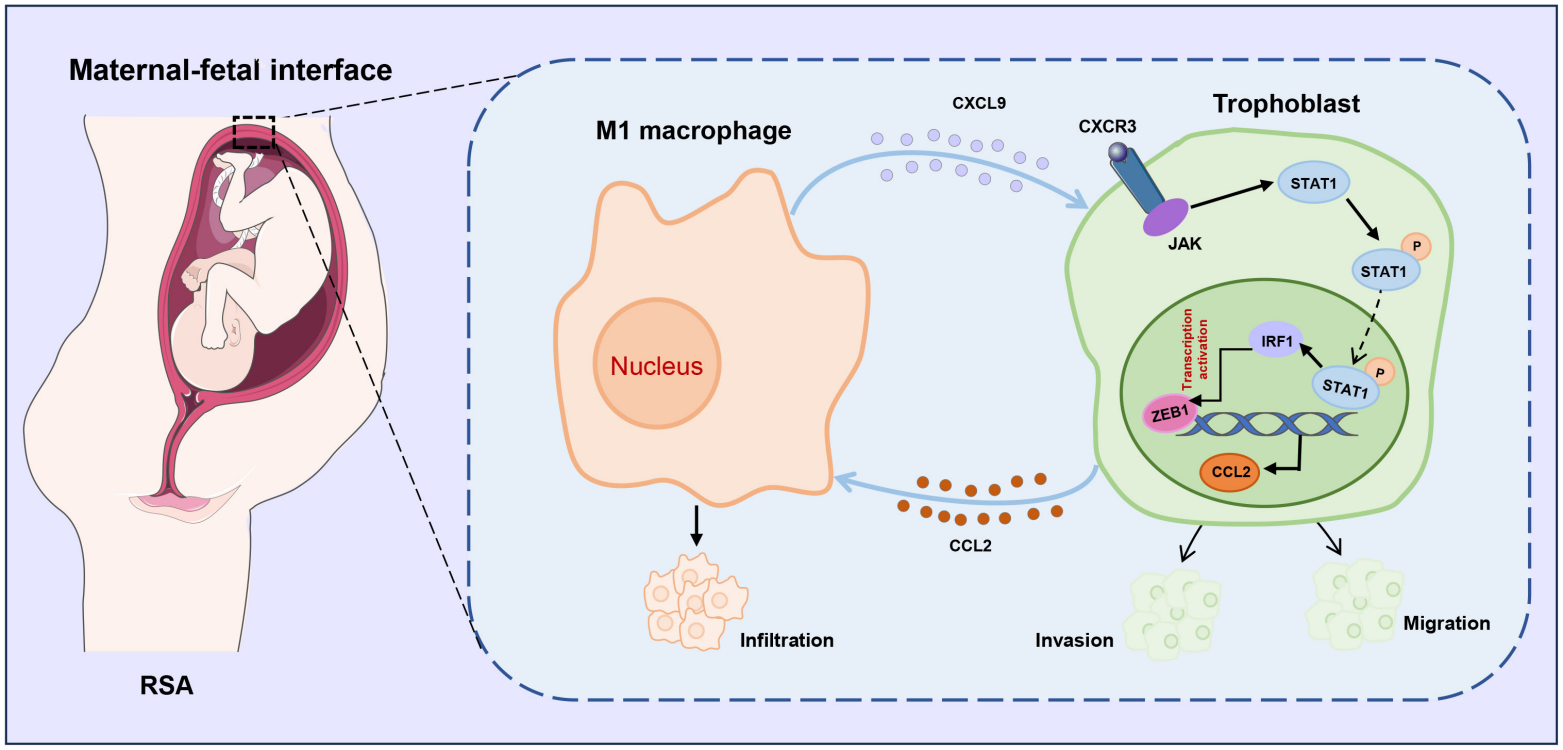
Schematic illustration of CXCL9/STAT1/ZEB1/CCL2 axis in M1 macrophage-trophoblast crosstalk. M1 macrophages provokes the release of CXCL9 and suppresses the migration and invasion of trophoblasts by regulating the JAK/STAT1/IRF1/ZEB1 axis, which in turn results in the production of CCL2 that promotes the recruitment of macrophages, implying a novel macrophage-trophoblast crosstalk at the maternal-fetal interface.

Accumulating evidence highlights the critical role of trophoblast-macrophage communication at the maternal-fetal interface in RSA (33, 34), providing a theoretical basis for elucidating the interactions between trophoblasts and immune cells in RSA. In this study, we demonstrated, for the first time that M1-Mφ stimulates the release of CXCL9, which activates the STAT1/IRF1 pathway, inhibits trophoblast EMT, invasion and migration, and suppresses ZEB1 expression. The resulting CCL2 upregulation further recruits macrophages and promotes the M1 polarization, thereby forming a positive feedback loop that disrupts placental development (**Figure 9**).

Mounting evidence from clinical and experimental studies has established that macrophages are essential decidual immune cells during pregnancy, and decidual macrophages are prone to M1 phenotype in patients with RSA compared to healthy controls (35). Therefore, we focused on the effects of M1-Mφ on trophoblast function and the underlying mechanisms. Although macrophages are known to modulate trophoblast function through paracrine signaling (36), we identified CXCL9 as the most responsive chemokine in M1-Mφ-trophoblast crosstalk. *In vitro* functional assays confirmed its pivotal role in regulating HTR-8 trophoblast cell migration and invasion. CXCL9, also known as monokine induced by gamma interferon (MIG), is mainly secreted by monocytes, fibroblasts and endothelial cells (37). It predominantly mediates immune cell infiltration and regulates tumor growth and metastasis (38). Emerging evidence suggests its involvement in the biological behavior of human chorionic trophoblast cells (39). More importantly, recent research has observed aberrant upregulation of CXCL9 in placenta tissues from spontaneous abortion patients compared to healthy pregnancies (40). Generally, CXCL9 binds to the G protein-coupled receptors or CXCR3, triggering downstream signaling. The CXCL9-CXCR3 axis typically activates endothelial cells and regulates the migration and invasion of tumor cells (41). Here, we found that the CXCL9-CXCR3 axis activates the JAK/STAT1 pathway, inhibiting trophoblast EMT, invasion, and migration *in vitro*.

The EMT process is orchestrated by core transcription factors, and ZEB1 has been identified as a key regulator of cellular invasion and migration in diverse biological processes (42, 43). Our investigation revealed that among EMT-related transcription factors, ZEB1 exhibited the most pronounced alteration in M1-Mφ-treated HTR-8 cells. The functional significance of this observation was confirmed through loss-of-function experiments, where ZEB1 knockdown effectively reversed M1-Mφ-induced inhibition of trophoblast EMT, invasion, and migration. These findings not only corroborate previous reports that established ZEB1 as a critical determinant of trophoblast invasiveness (44), but also highlight the novel role of ZEB1 in immune-trophoblast interactions. Building upon existing evidence of macrophage-mediated cytotoxicity in spontaneous abortion (45), our research elucidates mechanistic insights by identifying a novel positive feedback loop: M1-Mφ-derived signals (notably CXCL9) suppress ZEB1 expression, resulting in CCL2 upregulation in trophoblasts, which recruits additional M1-Mφ to the maternal-fetal interface. This observation aligns with clinical observations showing elevated CCL2 levels in RSA patients compared to normal pregnancies (46). Furthermore, elevated CCL2 may further promote macrophage recruitment and M1 polarization, leading to secretion of additional pro-inflammatory factors that impair trophoblast proliferation and invasion (47, 48).

IRF1, a transcription factor downstream of JAK-STAT1 signaling, is activated by STAT1 (49). Recent studies indicate that IRF1 is involved in cell proliferation, apoptosis, and is associated with EMT, invasion and migration in tumor cells (50, 51). In this study, we identified IRF1 as a novel suppressor of trophoblasts EMT, invasion, and migration through direct inhibition of ZEB1 transcription. Cumulatively, our findings highlight the STAT1/IRF1/ZEB1 axis as a critical regulator of trophoblast behavior. *In vivo*, anti-CXCL9 treatment alleviated embryo resorption rates,promoted trophoblast EMT, migration and invasion, and diminished macrophage recruitment. These results underscore the therapeutic potential of targeting CXCL9 in RSA management. Several limitations should be noted. Although the LPS-induced abortion model demonstrated the effects of CXCL9 inhibition on embryo absorption, the systemic inflammatory effects of LPS may introduce confounding variables. Therefore, further research should employ primary cells and alternative models to validate our findings. Additionally, targeting CXCL9 may cause systemic immunosuppression and compensatory chemokine activation; therefore, our future work will develop uterine-targeted nanoparticle delivery systems for CXCR3 antagonists to overcome these limitations.

In conclusion, our work elucidates a novel mechanism of macrophage-trophoblast crosstalk at the maternal-fetal interface, demonstrating its essential role in regulating trophoblast EMT, migration and invasion. Importantly, we identify CXCL9 as the pivotal molecular mediator orchestrating this pathological interaction, thereby offering a promising therapeutic target for RSA. These findings not only advance our understanding of pregnancy complications but also pave the way for developing novel immunomodulatory strategies in RSA treatment.

## Data Availability

The original contributions presented in the study are included in the article/**Supplementary Material**. Further inquiries can be directed to the corresponding authors.

## References

[1] El Hachem H Crepaux V May-Panloup P Descamps P Legendre G Bouet PE . Recurrent pregnancy loss: current perspectives. Int J Womens Health. (2017) 9:331–45. doi: 10.2147/IJWH.S100817, PMID: 28553146 PMC5440030

[2] Christiansen OB . Special issue recurrent pregnancy loss: etiology, diagnosis, and therapy. J Clin Med. (2021) 10. doi: 10.3390/jcm10215040, PMID: 34768559 PMC8584478

[3] Dimitriadis E Menkhorst E Saito S Kutteh WH Brosens JJ . Recurrent pregnancy loss. Nat Rev Dis Primers. (2020) 6:98. doi: 10.1038/s41572-020-00228-z, PMID: 33303732

[4] de Ziegler D Frydman RF . Recurrent pregnancy losses, a lasting cause of infertility. Fertil Steril. (2021) 115:531–2. doi: 10.1016/j.fertnstert.2020.12.004, PMID: 33581853

[5] Chen J Li Q Liu X Lin F Jing Y Yang J . Potential biomarkers and immune infiltration linking endometriosis with recurrent pregnancy loss based on bioinformatics and machine learning. Front Mol Biosci. (2025) 12:1529507. doi: 10.3389/fmolb.2025.1529507, PMID: 39963268 PMC11830612

[6] Alghetaa H Mohammed A Singh NP Bloomquist RF Chatzistamou I Nagarkatti M . Estrobolome dysregulation is associated with altered immunometabolism in a mouse model of endometriosis. Front Endocrinol (Lausanne). (2023) 14:1261781. doi: 10.3389/fendo.2023.1261781, PMID: 38144564 PMC10748389

[7] Harris LK Benagiano M D'Elios MM Brosens I Benagiano G . Placental bed research: II. Functional and immunological investigations of the placental bed. Am J Obstet Gynecol. (2019) 221:457–69. doi: 10.1016/j.ajog.2019.07.010, PMID: 31288009

[8] Kokkinos MI Murthi P Wafai R Thompson EW Newgreen DF . Cadherins in the human placenta–epithelial-mesenchymal transition (EMT) and placental development. Placenta. (2010) 31:747–55. doi: 10.1016/j.placenta.2010.06.017, PMID: 20659767

[9] Li Q Sharkey A Sheridan M Magistrati E Arutyunyan A Huhn O . Human uterine natural killer cells regulate differentiation of extravillous trophoblast early in pregnancy. Cell Stem Cell. (2024) 31:181–195.e9. doi: 10.1016/j.stem.2023.12.013, PMID: 38237587

[10] Krop J Tian X van der Hoorn ML Eikmans M . The mac is back: the role of macrophages in human healthy and complicated pregnancies. Int J Mol Sci. (2023) 24. doi: 10.3390/ijms24065300, PMID: 36982375 PMC10049527

[11] Colamatteo A Fusco C Micillo T D'Hooghe T Candia P Alviggi C . Immunobiology of pregnancy: from basic science to translational medicine. Trends Mol Med. (2023) 29:711–25. doi: 10.1016/j.molmed.2023.05.009, PMID: 37331882

[12] Wang XQ Li DJ . The mechanisms by which trophoblast-derived molecules induce maternal-fetal immune tolerance. Cell Mol Immunol. (2020) 17:1204–7. doi: 10.1038/s41423-020-0460-5, PMID: 32678309 PMC7784871

[13] Vondra S Hobler AL Lackner AI Raffetseder J Mihalic ZN Vogel A . The human placenta shapes the phenotype of decidual macrophages. Cell Rep. (2023) 42:111977. doi: 10.1016/j.celrep.2022.111977, PMID: 36640334

[14] Shimada S Ebina Y Iijima N Deguchi M Yamada H . Decidual CD68(+) HLA-DR(+) CD163(-) M1 macrophages increase in miscarriages with normal fetal chromosome. Am J Reprod Immunol. (2018) 79. doi: 10.1111/aji.12791, PMID: 29197148

[15] Wang L Wang H Luo J Xie T Mor G Liao A . Decorin promotes decidual M1-like macrophage polarization via mitochondrial dysfunction resulting in recurrent pregnancy loss. Theranostics. (2022) 12:7216–36. doi: 10.7150/thno.78467, PMID: 36438479 PMC9691373

[16] Yan S Ding J Wang Z Zhang F Li J Zhang Y . CTRP6 regulates M1 macrophage polarization via the PPAR-gamma/NF-kappaB pathway and reprogramming glycolysis in recurrent spontaneous abortion. Int Immunopharmacol. (2023) 124:110840. doi: 10.1016/j.intimp.2023.110840, PMID: 37696144

[17] Zhu X Liu H Zhang Z Wei R Zhou X Wang Z . MiR-103 protects from recurrent spontaneous abortion via inhibiting STAT1 mediated M1 macrophage polarization. Int J Biol Sci. (2020) 16:2248–64. doi: 10.7150/ijbs.46144, PMID: 32549769 PMC7294935

[18] Shang Y Wu S Li S Qin X Chen J Ding J . Downregulation of EZH2 in trophoblasts induces decidual M1 macrophage polarization: a potential cause of recurrent spontaneous abortion. Reprod Sci. (2022) 29:2820–8. doi: 10.1007/s43032-021-00790-1, PMID: 34820775 PMC9537223

[19] Ding J Zhang Y Cai X Zhang Y Yan S Wang J . Extracellular vesicles derived from M1 macrophages deliver miR-146a-5p and miR-146b-5p to suppress trophoblast migration and invasion by targeting TRAF6 in recurrent spontaneous abortion. Theranostics. (2021) 11:5813–30. doi: 10.7150/thno.58731, PMID: 33897883 PMC8058722

[20] Orecchioni M Ghosheh Y Pramod AB Ley K . Macrophage Polarization: Different Gene Signatures in M1(LPS+) vs. Classically and M2(LPS-) vs. Alternatively Activated Macrophages. Front Immunol. (2019) 10:1084. doi: 10.3389/fimmu.2019.01084, PMID: 31178859 PMC6543837

[21] Yan S Ding J Zhang Y Wang J Zhang S Yin T . C1QTNF6 participates in the pathogenesis of PCOS by affecting the inflammatory response of granulosa cellsdouble dagger. Biol Reprod. (2021) 105:427–38. doi: 10.1093/biolre/ioab094, PMID: 33959757

[22] Yao Y Xu XH Jin L . Macrophage polarization in physiological and pathological pregnancy. Front Immunol. (2019) 10:792. doi: 10.3389/fimmu.2019.00792, PMID: 31037072 PMC6476302

[23] Tokunaga R Zhang W Naseem M Puccini A Berger MD Soni S . CXCL9, CXCL10, CXCL11/CXCR3 axis for immune activation - A target for novel cancer therapy. Cancer Treat Rev. (2018) 63:40–7. doi: 10.1016/j.ctrv.2017.11.007, PMID: 29207310 PMC5801162

[24] Wang Z Xu H Chen M Lu Y Zheng L Ma L . CCL24/CCR3 axis plays a central role in angiotensin II-induced heart failure by stimulating M2 macrophage polarization and fibroblast activation. Cell Biol Toxicol. (2023) 39:1413–31. doi: 10.1007/s10565-022-09767-5, PMID: 36131165 PMC10425496

[25] Qiao M Li S Yuan J Ren W Shang Y Wang W . Delamanid suppresses CXCL10 expression via regulation of JAK/STAT1 signaling and correlates with reduced inflammation in tuberculosis patients. Front Immunol. (2022) 13:923492. doi: 10.3389/fimmu.2022.923492, PMID: 36426362 PMC9679411

[26] Shi D Li Y Shi X Yao M Wu D Zheng Y . Transcriptional expression of CXCL10 and STAT1 in lupus nephritis and the intervention effect of triptolide. Clin Rheumatol. (2023) 42:539–48. doi: 10.1007/s10067-022-06400-y, PMID: 36374433 PMC9873713

[27] Lamouille S Xu J Derynck R . Molecular mechanisms of epithelial-mesenchymal transition. Nat Rev Mol Cell Biol. (2014) 15:178–96. doi: 10.1038/nrm3758, PMID: 24556840 PMC4240281

[28] Park J Kim J Park B Yang KM Sun EJ Tognon CE . Novel identification of STAT1 as a crucial mediator of ETV6-NTRK3-induced tumorigenesis. Oncogene. (2018) 37:2270–84. doi: 10.1038/s41388-017-0102-2, PMID: 29391602

[29] Platanitis E Decker T . Regulatory networks involving STATs, IRFs, and NFkappaB in inflammation. Front Immunol. (2018) 9:2542. doi: 10.3389/fimmu.2018.02542, PMID: 30483250 PMC6242948

[30] Wang S Sun F Han M Liu Y Zou Q Wang F . Trophoblast-derived hyaluronan promotes the regulatory phenotype of decidual macrophages. Reproduction. (2019) 157:189–98. doi: 10.1530/REP-18-0450, PMID: 30605433

[31] Paparini DE Choudhury RH Vota DM Karolczak-Bayatti M Finn-Sell S Grasso EN . Vasoactive intestinal peptide shapes first-trimester placenta trophoblast, vascular, and immune cell cooperation. Br J Pharmacol. (2019) 176:964–80. doi: 10.1111/bph.14609, PMID: 30726565 PMC6433651

[32] Lin Z Shi JL Chen M Zheng ZM Li MQ Shao J . CCL2: An important cytokine in normal and pathological pregnancies: A review. Front Immunol. (2022) 13:1053457. doi: 10.3389/fimmu.2022.1053457, PMID: 36685497 PMC9852914

[33] Zhao X Jiang Y Luo S Zhao Y Zhao H . Intercellular communication involving macrophages at the maternal-fetal interface may be a pivotal mechanism of URSA: a novel discovery from transcriptomic data. Front Endocrinol (Lausanne). (2023) 14:973930. doi: 10.3389/fendo.2023.973930, PMID: 37265689 PMC10231036

[34] Liu Z Tang Y Zhang X Pei J Wang C Liu H . Crosstalk between placental trophoblast and decidual immune cells in recurrent miscarriage. Int J Med Sci. (2023) 20:1174–88. doi: 10.7150/ijms.86533, PMID: 37575278 PMC10416716

[35] Zhao QY Li QH Fu YY Ren CE Jiang AF Meng YH . Decidual macrophages in recurrent spontaneous abortion. Front Immunol. (2022) 13:994888. doi: 10.3389/fimmu.2022.994888, PMID: 36569856 PMC9781943

[36] Ding J Zhang Y Cai X Diao L Yang C Yang J . Crosstalk between trophoblast and macrophage at the maternal-fetal interface: current status and future perspectives. Front Immunol. (2021) 12:758281. doi: 10.3389/fimmu.2021.758281, PMID: 34745133 PMC8566971

[37] Leighton SP Nerurkar L Krishnadas R Johnman C Graham GJ Cavanagh J . Chemokines in depression in health and in inflammatory illness: a systematic review and meta-analysis. Mol Psychiatry. (2018) 23:48–58. doi: 10.1038/mp.2017.205, PMID: 29133955 PMC5754468

[38] Gorbachev AV Kobayashi H Kudo D Tannenbaum CS Finke JH Shu S . CXC chemokine ligand 9/monokine induced by IFN-gamma production by tumor cells is critical for T cell-mediated suppression of cutaneous tumors. J Immunol. (2007) 178:2278–86. doi: 10.4049/jimmunol.178.4.2278, PMID: 17277133

[39] Hanna J Wald O Goldman-Wohl D Prus D Markel G Gazit R . CXCL12 expression by invasive trophoblasts induces the specific migration of CD16- human natural killer cells. Blood (2003) 102:569–77. doi: 10.1182/blood-2003-02-0517, PMID: 12730110

[40] Spathakis M Filidou E Pappa C Arzou BC Georgiadis A Kontomanolis EN . Spontaneous abortion is associated with differentially expressed angiogenic chemokines in placenta and decidua. Arch Gynecol Obstet. (2023) 308:821–30. doi: 10.1007/s00404-022-06725-8, PMID: 35997970

[41] Li Z Liu J Li L Shao S Wu J Bian L . Corrigendum] Epithelial mesenchymal transition induced by the CXCL9/CXCR3 axis through AKT activation promotes invasion and metastasis in tongue squamous cell carcinoma. Oncol Rep. (2021) 45:791–2. doi: 10.3892/or.2020.7883, PMID: 33416176

[42] Jiang M Jike Y Liu K Gan F Zhang K Xie M . Exosome-mediated miR-144-3p promotes ferroptosis to inhibit osteosarcoma proliferation, migration, and invasion through regulating ZEB1. Mol Cancer. (2023) 22:113. doi: 10.1186/s12943-023-01804-z, PMID: 37461104 PMC10351131

[43] Guo Y Lu X Chen Y Clark G Trent J Cuatrecasas M . Opposing roles of ZEB1 in the cytoplasm and nucleus control cytoskeletal assembly and YAP1 activity. Cell Rep. (2022) 41:111452. doi: 10.1016/j.celrep.2022.111452, PMID: 36198275

[44] Lin L Lian X Liu Y Lin A . MicroRNA-574-5p affects trophoblast proliferation, migration and invasion by targeting ZEB1 in preeclampsia. Panminerva Med. (2022) 64:417–8. doi: 10.23736/S0031-0808.19.03833-3, PMID: 32000464

[45] True H Blanton M Sureshchandra S Messaoudi I . Monocytes and macrophages in pregnancy: The good, the bad, and the ugly. Immunol Rev. (2022) 308:77–92. doi: 10.1111/imr.13080, PMID: 35451089 PMC10317112

[46] Namli Kalem M Akgun N Kalem Z Bakirarar B Celik T . Chemokine (C-C motif) ligand-2 (CCL2) and oxidative stress markers in recurrent pregnancy loss and repeated implantation failure. J Assist Reprod Genet. (2017) 34:1501–6. doi: 10.1007/s10815-017-0992-5, PMID: 28707148 PMC5700001

[47] Wu Z Wang M Liang G Jin P Wang P Xu Y . Pro-inflammatory signature in decidua of recurrent pregnancy loss regardless of embryonic chromosomal abnormalities. Front Immunol. (2021) 12:772729. doi: 10.3389/fimmu.2021.772729, PMID: 34956198 PMC8694032

[48] Chen P Zhou L Chen J Lu Y Cao C Lv S . The immune atlas of human deciduas with unexplained recurrent pregnancy loss. Front Immunol. (2021) 12:689019. doi: 10.3389/fimmu.2021.689019, PMID: 34168655 PMC8218877

[49] Chen Z Yao MW Shen ZL Li SD Xing W Guo W . Interferon-gamma and tumor necrosis factor-alpha synergistically enhance the immunosuppressive capacity of human umbilical-cord-derived mesenchymal stem cells by increasing PD-L1 expression. World J Stem Cells. (2023) 15:787–806. doi: 10.4252/wjsc.v15.i8.787, PMID: 37700823 PMC10494569

[50] Rundberg Nilsson AJS Xian H Shalapour S Cammenga J Karin M . IRF1 regulates self-renewal and stress responsiveness to support hematopoietic stem cell maintenance. Sci Adv. (2023) 9:eadg5391. doi: 10.1126/sciadv.adg5391, PMID: 37889967 PMC10610924

[51] Zhang Y Zhang J Feng D Zhou H Gui Z Zheng M . IRF1/ZNF350/GPX4-mediated ferroptosis of renal tubular epithelial cells promote chronic renal allograft interstitial fibrosis. Free Radic Biol Med. (2022) 193:579–94. doi: 10.1016/j.freeradbiomed.2022.11.002, PMID: 36356714

