# Supplementary material for: Reciprocal activation between M1 macrophages and trophoblasts through CXCL9/STAT1/ZEB1/CCL2 axis promotes recurrent spontaneous abortion
Source: Front Immunol. 2025 Nov 7;16:1629370. doi: 10.3389/fimmu.2025.1629370 (PMC12634548; doi:10.3389/fimmu.2025.1629370)
Supplement: Supplementary file 1 [file DataSheet1.docx]

**Supplementary material**

**Supplementary Figure S1**


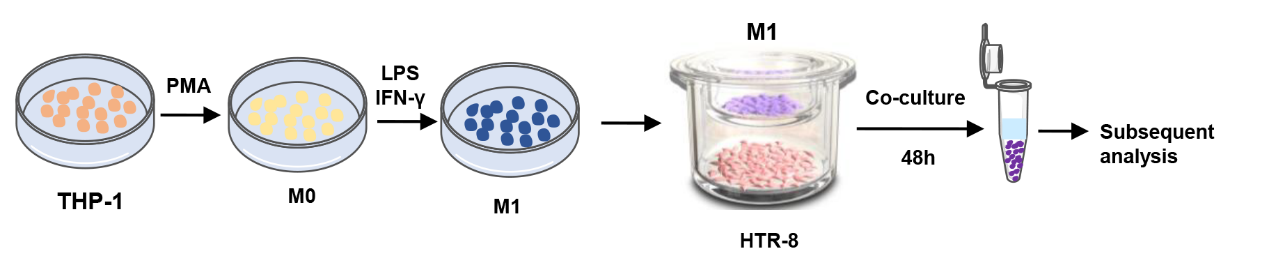


**Supplementary Figure S1.** Schema of THP-1-derievd M1 macrophage-HTR-8 co-culture model.

**Supplementary Figure S2**


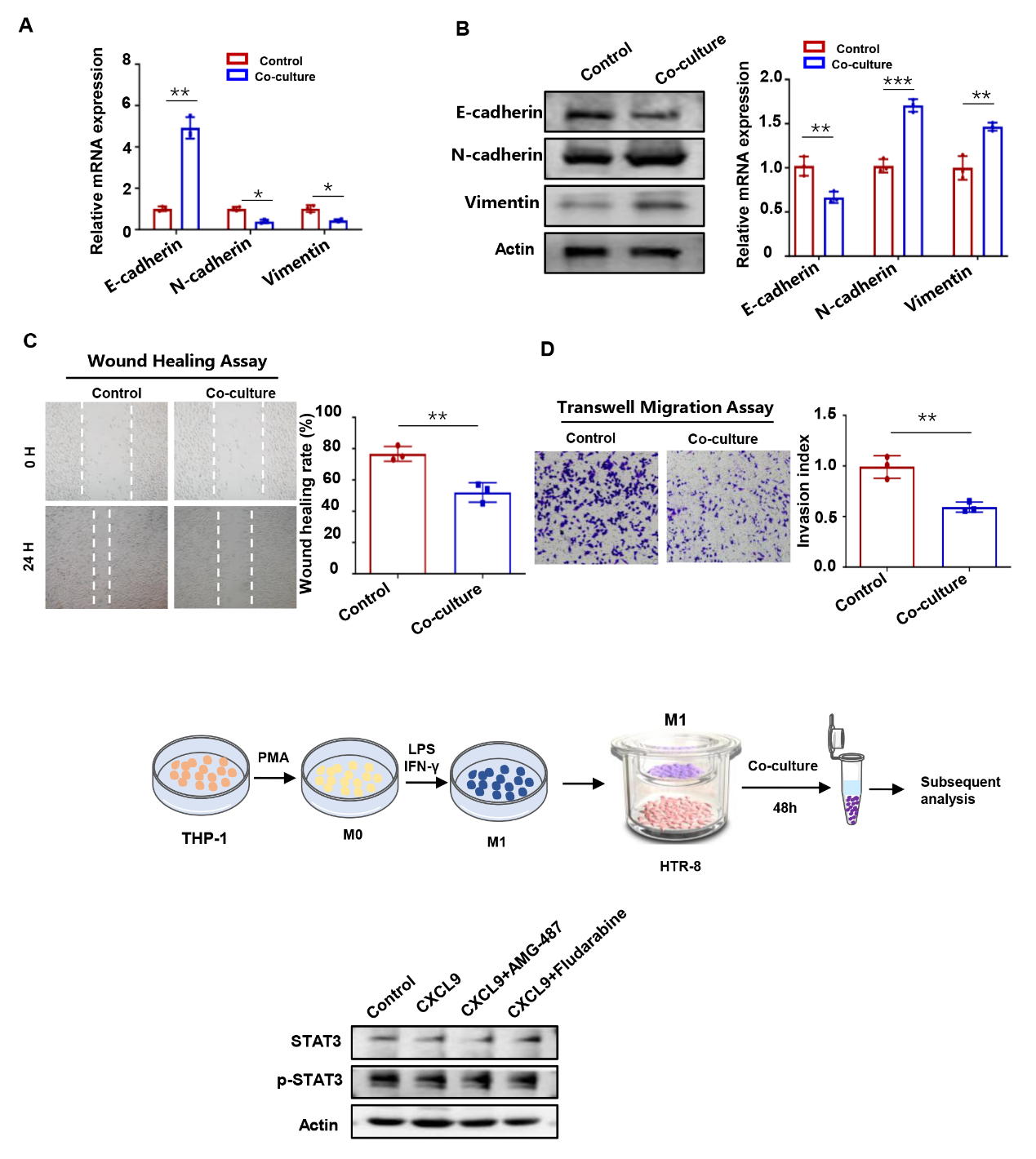


**Supplementary Figure S2. M1-Mφ impairs trophoblasts invasion and migration.** (A-B) qPCR and Western blot analysis of EMT markers (E-cadherin, N-cadherin, Vimentin) in HTR-8 cells following M1-Mφ coculture. (C, D) Migration and invasion of HTR-8 cells were determined by wound healing and transwell assays, respectively.

**Supplementary Figure S3**


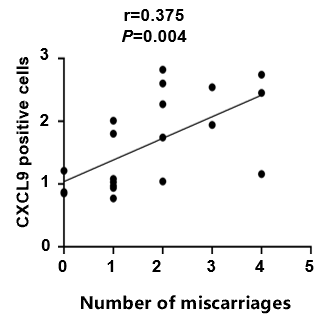


**Supplementary Figure S3.** The correlation between CXCL9 protein level and miscarriage history in normal and RSA patients.

**Supplementary Figure S4**


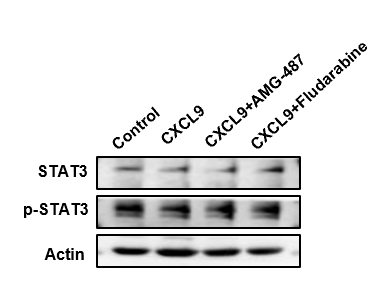


**Supplementary Figure S4.** Western blot analysis p-STAT3/STAT3 in HTR-8 cells alone, CXCL9 treatment HTR-8 cells, CXCL9+AMG-487 HTR-8 cells, and CXCL9+Fludarabine HTR-8 cells.

**Supplementary Tables**

**Supplementary Table S1.**  The roles of STAT1, CXCL9, CCL2 and ZEB1 in immunological responses.

| Molecule | Primary Role |
| --- | --- |
| STAT1 | IFN signaling transduction |
| CXCL9 | Chemokine (CXCR3 ligand) |
| CCL2 | Monocyte recruitment |
| ZEB1 | EMT transcription factor |

**Supplementary Table S2.** Baseline characteristics of the study population (n=10/group).

| Characteristics | Control group | RSA group |
| --- | --- | --- |
| Maternal age (y) | 28.3±2.9 | 30.9±3.2 |
| Body mass index (kg/m^2^) | 21.3±2.4 | 21.8±3.6 |
| Gestation age (weeks) | 8.2±1.3 | 8.8±1.8 |
| Number of miscarriages | 0.7±0.3 | 2.8±0.9** |
| Number of live births | 1.7±0.4 | 0.0±0.0* |

RSA, recurrent spontaneous abortion. Data are presented as the mean ± SD. ^*^, *P*＜0.05, ^**^, *P*＜0.01 vs control group.

**Supplementary Table S3.**The siRNA sequences used in this study.

| Gene | Sequence |
| --- | --- |
| STAT1 siRNA | 5′-CGAGAGCUGUCUAGGUUAAC-3′ |
| ZEB1 siRNA | 5′-CCGGTGTCTCCCATAAGTATCAATTCTCG-3′ |
| IRF1 siRNA | 5′-CCAGCGACCUGUACAACUU-3′ |

**Supplementary Table S4.** Sequences of primers used for qRT-PCR in this study.

| Gene | Sequence | |
| --- | --- | --- |
| CXCL9 | Forward | 5′- CCAGTAGTGAGAAAGGGTCGC-3′ |
|  | Reverse | 5′- AGGGCTTGGGGCAAATTGTT-3′ |
| CXCL10 | Forward | 5′-GTGGCATTCAAGGAGTACCTC-3′ |
|  | Reverse | 5′-TGATGGCCTTCGATTCTGGATT-3′ |
| TNF-α | Forward | 5′-GAGGCCAAGCCCTGGTATG-3′ |
|  | Reverse | 5′-CGGGCCGATTGATCTCAGC-3′ |
| TGF-β | Forward | 5′-CAATTCCTGGCGATACCTCAG-3′ |
|  | Reverse | 5′-GCACAACTCCGGTGACATCAA-3′ |
| CCL18 | Forward | 5′-CTCTGCTGCCTCGTCTATACCT-3′ |
|  | Reverse | 5′-CTTGGTTAGGAGGATGACACCT-3′ |
| IL-6 | Forward | 5′-GTGGCATTCAAGGAGTACCTC-3′ |
|  | Reverse | 5′-TGATGGCCTTCGATTCTGGATT-3′ |
| IL-1β | Forward | 5′-GTGGCATTCAAGGAGTACCTC-3′ |
|  | Reverse | 5′-TTTGAAGTTGACGGACCCCA-3′ |
| IL-10 | Forward | 5′-GACTTTAAGGGTTACCTGGGTTG-3′ |
|  | Reverse | 5′-TCACATGCGCCTTGATGTCTG-3′ |
| E-cadherin | Forward | 5′-CTACAATGAGCTGCGTGTGG-3′ |
|  | Reverse | 5′-AGGTCCAGACGCAGGATGGC-3′ |
| N-cadherin | Forward | 5′-CAGTATCCGGTCCGATCTGC-3′ |
|  | Reverse | 5′-GTCCTGCTCACCACCACTAC-3′ |
| Vimentin | Forward | 5′-GACGCCATCAACACCGAGTT-3′ |
|  | Reverse | 5′-CTTTGTCGTTGGTTAGCTGGT-3′ |
| Snail | Forward | 5′-TCTGAGGCCAAGGATCTCCA-3′ |
|  | Reverse | 5′-TGGCTTCGGATGTGCATCTT-3′ |
| FOXQ1 | Forward | 5′-TGATTTCTTGCTATTGACCGATGC-3′ |
|  | Reverse | 5′-GCCCAAGGAGACCACAGTTAGAG-3′ |
| HMGA2 | Forward | 5′-ACCCAGGGGAAGACCCAAA-3′ |
|  | Reverse | 5′-CCTCTTGGCCGTTTTTCTCCA-3′ |
| Twist1 | Forward | 5′-TCTACCAGGTCCTCCAGAGC-3′ |
|  | Reverse | 5′-CTCCATCCTCCAGACCGAGA-3′ |
| ZEB1 | Forward | 5′-CAACTACGGTCAGCCCT-3′ |
|  | Reverse | 5′-GCGGTGTAGAATCAGAGTC-3′ |
| Actin | Forward | 5′-GAATTCATGTTTGAGACCTTCAA-3′ |
|  | Reverse | 5′-CCGGATCCATCTCTTGCTCGAAGTCCA-3′ |
| IRF9 | Forward | 5′-GCCTGTAACACACTGCCTCT-3′ |
|  | Reverse | 5′-CTTGTAGGGCTCAGCAACAT-3′ |
| IRF1 | Forward | 5′-GACCCTGACCCGAAAACCT-3′ |
|  | Reverse | 5′-TGCTCCTGCTTCTTGCTT-3′ |
| TAP1 | Forward | 5′-GCAGTCAACTCCTGGACCACTA-3′ |
|  | Reverse | 5′-CAAGGTTCCCACTGCTTACAGC-3′ |
| H-iNOS | Forward | 5′-TTCAGTATCACAACCTCAGCAAG-3′ |
|  | Reverse | 5′-TGGACCTGCAAGTTAAAATCCC-3′ |
| H-CD86 | Forward | 5′-CTGCTCATCTATACACGGTTACC-3′ |
|  | Reverse | 5′-GGAAACGTCGTACAGTTCTGTG-3′ |
| H-TNF-α | Forward | 5′-TCTCGAACCCCGAGTGACAA-3′ |
|  | Reverse | 5′-TGAAGAGGACCTGGGAGTAG-3′ |
| CCL2 | Forward | 5′-CAGCCAGATGCAATCAATGCC-3′ |
|  | Reverse | 5′-TGGAATCCTGAACCCACTTCT-3′ |

**Supplementary Table S5:** Sequences of primers for chromatin-immunoprecipitation (ChIP).

|  | **Primer Sequences** | **Product length (bp)** | **Product sites** |
| --- | --- | --- | --- |
| Site1 | 1F: TTTCTCCCTCCCCTCTGGGA  1R: AAAAATATAATTATGGATTG | 75 | -317 to -391 |
| Site2 | 2F: TTCCTGTCTAGAAGCAGATA  2R: TCTTTAAAATGCAAGTGTTTA | 75 | -242 to -316 |
| Site3 | 3F: AATATATTCGAGCCATCATT  3R: TCCACTCCTTGCTATAACAA | 76 | -166 to -241 |
| Site4 | 4F: CGTCTGTTGATTATAAACGA  4R: AAAGCCACATCAGCAACAGCG | 165 | -1 to -165 |
